# Perceived health benefits of martial arts and combat sports

**DOI:** 10.3389/fpsyg.2026.1774069

**Published:** 2026-04-20

**Authors:** Erika Zormann, Markus Foramitti, Urs Markus Nater

**Affiliations:** 1 Department of Clinical and Health Psychology, Faculty of Psychology, University of Vienna, Vienna, Austria; 2 Research Platform “The Stress of Life (SOLE) - Processes and Mechanisms Underlying Everyday Life Stress”, University of Vienna, Vienna, Austria

**Keywords:** combat sports, HRQOL, martial arts, mental health, perceived health benefits, physical health

## Abstract

**Introduction:**

While there are many anecdotal claims and a growing body of literature on the health benefits of martial arts (MA) and combat sports (CS), research on their perception by practitioners is scarce.

**Methods:**

This cross-sectional study (*N* = 268) explored the associations between self-rated health and perceived health benefits of MA/CS, by utilizing a MA/CS specific questionnaire as well as widely used measures for mental and physical health. Additionally, the association of training age (in years) and training frequency (per month) with perceived health benefits was examined.

**Results:**

The results of the hierarchical multiple linear regression analyses indicated that training frequency was not associated with perceived health benefits, whereas training age showed a small association. Interestingly, in an explorative step, current self-rated health was associated with additional variance in the perceived health benefits. Small to medium associations were found between mental and physical health.

**Discussion:**

It is concluded that health benefits are reported by MA/CS practitioners regardless of training age or frequency, and may therefore be relevant to a larger population. Nevertheless, future studies are needed to further explore these relationships using a longitudinal approach.

## 1 Introduction

Given the generally recognized benefits of physical activity for both mental and physical health, it is reasonable to implement programs that promote increased physical activity in everyday life, as recommended by World Health Organization (2020). In addition to its health protective quality, research also illustrates positive effects of physical activity, when it is specifically utilized as a mental health intervention—both as a complement to standard treatment and as an alternative when conventional treatment is unavailable or not desired by patients (Kvam et al., 2016; Richardson et al., 2005). This is especially true with respect to depressive symptoms, which improve significantly in healthy and clinical populations after physical exercise (Recchia et al., 2023; Schuch et al., 2016). This effect appears to be the result of enhanced immune function and complex physiological processes, including those involved in stress responses (Mikkelsen et al., 2017; Salmon, 2001), which are linked to (neuro-)inflammatory processes and depression (Hassamal, 2023; Wirtz and von Känel, 2017). Furthermore, psychological mechanisms of physical activity, such as distraction, improved self-efficacy, and emotion regulation, as well as engaging in social interaction, have a positive impact on mental health (Bernstein and McNally, 2018; Mikkelsen et al., 2017; Paluska and Schwenk, 2000).

*Martial arts* (MA) and *combat sports* (CS) encompass a wide range of combat training systems that vary significantly in their development of physical and mental skills, cultivation of mindfulness, and spiritual or philosophical insight. In Western countries, they are primarily practiced for recreational, self-defense, or competitive purposes. MA/CS can also be divided into “hard” and “soft” styles, mainly differing in their extent of physical confrontation (Bu et al., 2010). In the present study, we adopt the classification proposed by (Ciaccioni et al., 2024), distinguishing “hard” styles, which emphasize direct striking techniques and forceful physical engagement, from “soft” styles, which rely more on redirection, control, and the use of an opponent’s momentum rather than direct impact. A significant advantage of this diversity is that MA/CS are suitable for individuals across various age groups and health conditions.

Research indicates that MA/CS practitioners have increased physical health, including better fitness levels, greater strength and flexibility (Origua Rios et al., 2018; Wehner et al., 2021), and better bone structure (Chow et al., 2018). Multiple cross-sectional studies found higher health-related quality of life and mental health scores among MA/CS practitioners of various styles compared to normative values or non-practitioners (e.g., Miyata et al., 2020). “Soft” styles, such as Tai Chi, can also be considered as mind–body interventions, because they incorporate mindful movements and breathing techniques. Literature on these interventions suggests a positive impact on reducing stress (Zhu et al., 2021) and depression (Galante et al., 2021). However, beneficial effects on chronic stress were also observed in “hard” MA/CS due to the combination of mindfulness and physical movement (Naves-Bittencourt et al., 2015). Review articles predominantly underscore the positive impact of diverse MA/CS on mental health: Not only “soft” MA/CS such as Tai Chi are associated with reductions in depression and stress (e.g., Wang et al., 2010), but also “hard” MA/CS such as Taekwondo, Judo, and Karate have been found to increase mood and decrease depression (e.g., Origua Rios et al., 2018). In a similar manner, when specifically implemented as a health intervention, MA/CS seem to be beneficial for wellbeing and depression (Moore et al., 2019).

Several aspects of MA/CS demonstrate contributions to mental health. Resilience is recognized as a protective factor against stress and depression (Marin et al., 2011) and has been found to improve with Karate training (Witte et al., 2016). Similarly, emotional regulation has been shown to benefit from MA/CS training (Ciaccioni et al., 2024). Cognitive functioning, another substantial facet of mental health, is closely related to stress and depression, although the direction of the effect is not clear (Marin et al., 2011; Wiels et al., 2020). Studies applying MA/CS interventions have found improvements in attention in both young and older adults (Converse et al., 2014; Witte et al., 2016), as well as increased memory in children (Li et al., 2023). Most reviews report an improvement in cognitive performance for both “soft” and “hard” MA/CS (e.g., Abbott and Lavretsky, 2013; Origua Rios et al., 2018). In contrast, a recent systematic review does not endorse “hard” MA/CS as an intervention for improving cognition in older adults due to the limited evidence (Ramos-Espinoza et al., 2024). Similarly, another systematic review provided evidence for only a few cognitive benefits but an unclear association with memory and attention in the adult population (Ciaccioni et al., 2024). Furthermore, social aspects of MA/CS are important for mental health. The feeling of social connectedness serves as a protective factor against mental illnesses, particularly depression, and modulates stress responses (Goodyke et al., 2022; Wickramaratne et al., 2022). For example, a randomized controlled trial with depressed older participants found that during a Tai Chi intervention, social support had a significant effect on reducing depressive symptoms (Cho, 2008). Furthermore, the results by Moradi et al. (2023) suggest a positive relationship between perceived social support and resilience in MA/CS practitioners. Another study indicated that Karate training improved social confidence in children (Conant et al., 2008). Overall, it is reasonable to assume that the multifaceted approach of MA/CS has beneficial effects on physical and mental health. However, methodological shortcomings and the diversity of styles and populations examined could play a significant role in ambiguous findings.

While research on the health advantages of MA/CS remains inconclusive, such benefits are frequently reported by practitioners themselves. In a qualitative survey, Fuller and Lloyd (2019) found that practitioners considered themselves happier, less anxious, and to have greater life satisfaction since engaging in MA/CS. Additionally, 80% of participants were convinced that the health benefits they experienced were directly related to practicing MA/CS, although confirmation bias may have influenced responses. Similarly, conscious changes toward a healthier lifestyle (e.g., eating well, improving one’s posture, reducing smoking) were made after starting MA/CS practice.

Nevertheless, there is a notable lack of quantitative research on perceived benefits, despite the fact that such studies could provide another important perspective on how practitioners interpret the contribution of MA/CS to their wellbeing.

Perceived benefits can serve as an indicator for understanding broader health outcomes. Given that patients’ medical decisions are also influenced by their perception of benefits (Singer et al., 2014), research in this area could additionally help inform strategies to enhance the effectiveness of health interventions. There is substantial evidence that self-rated health is positively associated with objective health outcomes, such as mortality or physical parameters (e.g., Kaplan and Camacho, 1983; Lorem et al., 2020). This relationship suggests that self-rated health is a reliable indicator of overall wellbeing. Based on this assumption, the present study investigates whether perceived health benefits of MA/CS align with practitioners’ self-rated health. However, because the present study is cross-sectional, directionality cannot be inferred, and reverse causality is plausible (e.g., individuals with better self-rated health may be more likely to initiate or maintain MA/CS practice). Therefore, all findings are interpreted as associations rather than causal effects.

Finally, although often not reported, it is considered important to investigate the role of training age and training frequency (Ciaccioni et al., 2024). This seems plausible, as the development of skills is accompanied by functional and structural adaptations in the brain (Chang, 2014). These neurobiological changes might underscore the findings that associate training age and training frequency with self-rated health outcomes (e.g., Chekroud et al., 2018; Stanton and Reaburn, 2014). Similarly, research on MA/CS practitioners has discovered that training age and training frequency are positively associated with mental health outcomes, emphasizing the potential of these variables to influence wellbeing through MA/CS training (Miyata et al., 2020).

Taken together, although evidence so far hints at positive effects, research on the perceived health benefits of MA/CS training is scarce. Therefore, this study aims to investigate whether MA/CS are perceived as health-promoting, considering the effects of training age and frequency. Another objective of this study is to explore the associations between self-rated health and perceived health benefits, offering insights into how individuals’ health perceptions impact their overall wellbeing. Thus, it is hypothesized that (1a) training age and (1b) frequency of MA/CS training have a positive influence on perceived benefits for mental health. Similarly, it is proposed that (1c) training age and (1d) frequency of MA/CS training have a positive impact on perceived benefits for physical health. Finally, it is expected that perceived health benefits through MA/CS training are positively associated with self-rated (2a) mental health and (2b) physical health.

## 2 Methods

### 2.1 Sample

The sample consisted of 268 participants, who were recruited directly via their MA/CS club, association, or online via MA/CS social media groups. Participants were required to be over 16 years of age, fluent in German, and currently practicing MA/CS in Austria or Germany. No minimum criteria for training age or frequency were set. The sample size for this study was calculated prior to data collection via G*Power 3.1 (Faul et al., 2007). The suggested minimum of 82 participants for two-tailed correlations (*ρ* = 0.30, 1 − *β* = 0.80, *α* = 0.05) was exceeded, allowing for a broad range of MA/CS.

### 2.2 Experimental design

The study employed a cross-sectional design to investigate the perceived health benefits and their relationships with physical and mental health among MA/CS practitioners. Data were collected using an online survey platform. Due to the specific group of interest, a non-random sampling method was carried out.

### 2.3 Measuring instruments and measured variables

All the following measurement instruments were provided to the participants in the German using the online survey tool Unipark (Tivian, 2024).

#### 2.3.1 Sociodemographic data

For the sample description, participants’ sociodemographic information was collected, including gender, age, nationality, and educational background.

#### 2.3.2 Martial arts and combat sports questionnaire (MACS-Q)

For this study, the MACS-Q was developed as part of a larger project to measure perceived health benefits of MA/CS practice. The items were generated based on literature review, feedback from stakeholder groups, and anecdotal evidence from practitioners. Experts in the field were consulted for feedback on the questionnaire. In the end, 53 items were formulated to assess the scales “training age,” “training frequency,” “initial motives,” “perceived health benefits”, and “training elements.” The original German items and the English translation can be found as an online Supplementary material. The current study only used the scales training frequency, training age, and perceived health benefits, with the latter consisting of four factors: physical, cognitive, emotional, and social benefits. Participants entered their MA/CS in a multiple-choice or open format. Training age (in years) and frequency (in sessions per month) were entered in an open format. The perceived health benefits (e.g., “My MA/CS training is contributing to that I am rarely ill”) are rated on a five-point Likert scale (i.e., 1 = “strongly disagree” to 5 = “strongly agree”). The scales for perceived health benefits consisted of the summed-up item scores for each factor. Cronbach’s alpha for the four factors was calculated and revealed good internal consistency for cognitive (*α* = 0.83), emotional (*α* = 0.85) and social benefits (*α* = 0.81), but poor internal consistency for physical benefits (*α* = 0.63). In this study, physical health benefits are represented by its respective factor, and mental health benefits consist of the three factors cognitive benefits, emotional benefits, and social benefits.

Given the limited internal consistency of the physical benefits subscale, exploratory item diagnostics were performed. Two items (“fitness” and “lifestyle”) showed weaker conceptual specificity and comparatively low factor representation. Removing either item alone had a negligible impact on internal consistency (*α* = 0.64 and *α* = 0.61, respectively), whereas removing both increased internal consistency slightly (*α* = 0.67). To preserve conceptual breadth, the original 5-item subscale was retained for the primary analyses. To examine whether this affected substantive conclusions, all primary regression and correlation analyses were repeated using the reduced 3-item version of the physical benefits scale as a sensitivity check. The pattern, effect directions, and statistical significance of the results remained unchanged.

#### 2.3.3 Patient health questionnaire depression scale (PHQ-9)

The PHQ-9 is part of the screening tool PHQ-D (Gräfe et al., 2004), which consists of 78 items assessing eight psychological disorders. Although participants responded to all items of the PHQ-D, this paper reports only the results of the nine-item depression subscale, PHQ-9. The questions refer to how often respondents experienced depressive symptoms (e.g., “feeling tired or having little energy”) over the past 2 weeks, using a 4-point Likert scale (i.e., 0 = “not at all” to 3 = “nearly every day”). The PHQ-9 demonstrated good construct validity and showed good reliability (*α* = 0.88) in its validation study. In the present study, the depression subscale represents a facet of mental health, which was constructed by summing up its scores, with higher scores indicating more severe symptoms.

#### 2.3.4 Perceived stress scale (PSS-10)

The PSS-10 (Klein et al., 2016) measures subjective stress by assessing how unpredictable, uncontrollable, and overwhelming situations in everyday life were perceived by the respondent over the past 4 weeks. The scale contains 10 items (e.g., “In the last month, how often have you felt that you were on top of things?”) and is rated on a five-point Likert scale from 0 (“*Never*”) to 4 (“*Very often*”). The scale for perceived stress is built by inverting positively formulated items and summing up all item scores, and ranges from 0 to 40. A higher score indicates a greater perceived stress level in the last month. Validity and good internal consistency (*α* = 0.84) could be demonstrated. The PSS was used as one of the measures for mental health in the present study.

#### 2.3.5 Short form health survey 36 (SF-36)

The SF-36 (Bullinger et al., 1995) is a broad standard measure for health-related quality of life (HrQoL). It consists of 36 items, of which 35 are part of the following eight subscales: physical functioning, physical role functioning, bodily pain, general health perception, vitality, social role functioning, emotional role functioning, and mental wellbeing. The first four and the latter four subscales can be subsumed into the physical component summary scale (PCS) and mental component summary scale (MCS). The response format consists of Likert scales and dichotomous (“Yes”/“No”) questions. The German version demonstrates satisfying psychometric properties, and the subscales had acceptable and good internal consistency (*α* = 0.73–0.88) in healthy populations, except for the subscale general health perception (*α* = 0.66–0.69). The scales were calculated using the standard MOS SF-36 scoring instructions (Ware, 1993). In the present study, the two main dimensions, PCS and MCS, were used to indicate self-rated physical and mental health, respectively. Unlike the other health questionnaires, higher scores indicate better health.

#### 2.3.6 Physical- and sports activity questionnaire (BSA-F)

The BSA-F (Fuchs et al., 2015) assesses physical activity across the sections of work life, leisure time, and sports participation over the past 4 weeks. The measure consists of 17 items with varying response formats, including four-point Likert scales (“*none*” to “*a lot*”) and open-ended questions for the number of days and minutes spent on specific activities (e.g., “walking to the grocery store”). Scores for leisure-time and sports activity can be summed up to a total activity score (in minutes per week). Although reliability measures could not be made due to the design of the validation studies, preliminary evidence demonstrated the validity of the BSA-F. In the present study, the sports activity scores were divided into MA/CS and other sports activities to examine the potential role of other sports in influencing the perception of health benefits. To calculate the index for *other sports activities*, the product of minutes and days spent on other sports was summed, divided by four, and then converted to hours.

### 2.4 Procedure

Overall, approximately 150 MA/CS clubs and associations located in Austria and Germany, as well as the University Sports Institute of the University of Vienna, were contacted via e-mail to help distribute the online survey to their members. Initially, only Viennese practitioners were contacted; however, due to the summer holiday season during which data collection took place, many clubs and participants were inaccessible. Therefore, data collection was extended to include the entirety of Austria and Germany. The survey was then additionally posted to Austrian and German MA/CS groups on social media. Clubs and associations distributed the survey via newsletters to their members and/or flyers in their training facilities. Data were collected at a single point in time over a six-week period, starting from the beginning of July 2024. After giving informed consent, respondents were able to participate in the survey. If the inclusion criteria were not met, participants were excluded at the beginning of the survey. The questionnaires were presented in the following order to all subjects: Sociodemographic data, MACS-Q, PSS, PHQ-D, SF-36, and BSA-F. The rationale was to present the MA/CS-specific questionnaire early on, to evoke interest and identification with the study, helping to minimize the risk of participants disengaging. It was possible to pause survey completion at any time and resume from the same page, using the same device. Due to the length of the survey, participants had to respond to attention-checking items (e.g., “Currently I am answering questions about…?”) to ensure attention was maintained. To maintain motivation, encouraging statements were presented in between questionnaires (e.g., “You demonstrate endurance and true team spirit! Now, we will continue with some questions about your health.”). At the end of the survey, participants had the opportunity to take part in a prize draw, where they could win vouchers for sports gear, nutritional supplements, or wellness treatments. Moreover, they were offered to be informed about the study results.

### 2.5 Analysis methods

The collected data was filtered for incomplete and implausible cases. The plausibility of the datasets was confirmed for participants who entered their MA/CS sports activity in the BSA-F, provided correct answers to all attention items, and met the minimum time criterion for completing the survey. This criterion was set to 10 min, as this time was approximately needed to quickly read through and understand all the items of the questionnaires, as indicated by pilot testing of the survey. Regarding the BSA-F, nominal statements had to be corrected to analogous quantitative values. Approximate statements (e.g., “60–90 minutes”) were adjusted to their numerical mean. Due to a programming error in the online survey, almost a third (31.34%) of the datasets showed unclear or missing values for the BSA leisure time activity. To avoid bias, a total activity score was not calculated.

All data analyses were performed using JASP version 0.19.0 (JASP Team, 2024). A confirmatory factor analysis was carried out using the SEM module with Maximum Likelihood estimation to validate the hypothesized scales for perceived health benefits in MA/CS. To improve model fit, modifications were made based on modification indices only if theoretical considerations supported these. Hierarchical multiple linear regressions with respective benefit as outcome variable were applied to examine the predictive effects of frequency and training age on perceived health benefits of MA/CS. In the first step, confounding variables (i.e., age, other sports activity) were included, while in the second step, either the predictor training age or training frequency was added. Although Scult et al. (2017) reported in their systematic review and meta-analysis that current depressive symptoms influenced the significance of the results, Askim and Knardahl (2021) found only limited differences in self-reported data based on participants’ affective states. To address the potential role of response bias in the current health state, a third step was added exploratively, incorporating the variables PCS and MCS. Assumption checks were conducted for each model. Partial regression plots and residuals vs. predicted plots were visually examined to verify the assumption of linearity and homoscedasticity of residuals, respectively. Multicollinearity was assessed via the VIF and tolerance statistics. A VIF of <10 and tolerance of >0.2 implied non-multicollinearity (Field, 2013). The Durbin-Watson test was used to assess the independence of residuals, with values close to 2 indicating no autocorrelation (Field, 2013). Q–Q plots of residuals were inspected to register normality of residuals. Additionally, influential cases were examined based on the indices of standardized residuals, Cook’s Distance, DFBETAS, leverage, and Mahalanobis. After excluding cases conspicuous in at least two indices, no significant changes were detected. Therefore, the reported results are based on the whole sample. Bootstrapping with 1,000 replicates was also used to estimate robust standard errors and bias-corrected accelerated 95% confidence intervals, as the assumptions were mildly violated.

To explore the associations between self-rated physical and mental health with perceived health benefits, a partial correlation was conducted. The assumption of normality was tested by visual inspection of distribution plots and the Shapiro–Wilk test for Multivariate Normality (*p* < 0.001), which both indicated non-normality. Consequently, the non-parametric Spearman’s rho statistic was used for more robust correlational results. The calculations were adjusted for age and other sports activities.

In additional exploratory analyses, participants were grouped by style type (“hard,” “soft,” or a combination of both, based on Ciaccioni et al., 2024); all training systems and their classification are presented in Appendix A to examine potential differences in perceived health benefits. Style type was tested both as a predictor of mean differences and as a moderator of the associations between training characteristics (training age and training frequency) and perceived benefits. Style type was entered as a categorical predictor using dummy coding with hard-style-only practitioners as the reference group. Group differences were therefore estimated within the regression framework via planned contrasts. Furthermore, initial training motives (health, fun, social contacts, self-defense, philosophy) were examined as predictors and as potential moderators by including main effects and interaction terms in the regression models. All analyses were adjusted for age, other sports activity, PCS, and MCS. For exploratory moderation analyses, training age and training frequency were mean-centered prior to forming interaction terms.

## 3 Results

### 3.1 Descriptive statistics

Table 1 summarizes the demographic characteristics of the sample, while MA/CS specific distributions are presented in Table 2. Other sports activity (*M* = 135.70, *SD* = 172.52) was mainly distributed at the lower end, with 50% engaging in other sports for less than 75 min/week, whereas the maximum reached 985 min/week. Table 3 presents the descriptive statistics and internal consistency for the scales. All benefit scales showed a trend toward being perceived as contributing to health. Intercorrelations were computed to assess the range of associations between the investigated variables (Table 4).

**Table 1:** Characteristics of respondents (*N* = 268).

Variables	*n*	%
Age; mean (SD)	40.44 (14.28)	
Gender		
Female	111	41.42
Male	155	57.84
Other	2	0.74
Nationality		
Austrian	183	68.28
German	62	23.13
Other	23	8.59
Education		
Primary school	1	0.37
Middle school	12	4.48
Dual professional training	23	8.58
High school	62	23.13
Advanced professional training	16	5.97
University (Bachelor)	39	14.55
University (Master)	93	34.70
University (PhD)	20	7.46
Other	2	0.75

**Table 2:** Distribution of MA/CS and training age (*N* = 268).

Variables	*n*	%
MA/CS^a^		
Aikido	16	5.97
Boxing	30	11.19
Brazilian Jiu Jitsu	26	9.70
Hapkido	8	2.99
Iaido	17	6.34
Jiu Jitsu	42	15.67
Judo	28	10.45
Karate	37	13.81
Kendo	8	2.99
Kickboxing	41	15.30
Krav Maga	15	5.60
Kung Fu	18	6.72
Mixed martial arts	5	1.87
Muay Thai	15	5.60
Ninjutsu	4	1.49
Taekwondo	43	16.05
Tai Chi	27	10.08
Training age		
≤6 months	9	3.36
7–47 months	58	21.64
≥48 months	201	75.00

a MA/CS percentage not cumulative (multiple selection of MA/CS was possible).

**Table 3:** Descriptives and internal consistency for assessed scales.

Scale	*M (SD)*	Cronbach’s *α*
MACS-Q physical benefits	20.53 (2.80)	0.63
MACS-Q emotional benefits	35.92 (5.71)	0.85
MACS-Q cognitive benefits	15.40 (3.26)	0.83
MACS-Q social benefits	15.75 (3.04)	0.81
PSS	13.38 (5.60)	0.81
PHQ-9	4.17 (3.36)	0.78
SF-36 physical functioning	96.38 (10.06)	0.88
SF-36 role-physical	92.91 (20.21)	0.80
SF-36 bodily pain	78.00 (15.70)	0.73
SF-36 general health	75.87 (14.50)	0.63
SF-36 vitality	61.75 (17.12)	0.85
SF-36 role-emotional	83.09 (29.78)	0.72
SF-36 social function	85.45 (21.57)	0.60
SF-36 mental health	77.64 (13.97)	0.76

**Table 4:** Intercorrelations for study variables (*ρ*).

Variable	1	2	3	4	5	6	7	8	9	10	11
MACS-Q PB	–										
MACS-Q CB	0.54***	–									
MACS-Q EB	0.60***	0.63***	–								
MACS-Q SB	0.40***	0.46***	0.62***	–							
PP	0.27***	0.26***	0.25***	0.25***	–						
TF	0.06	0.15*	0.08	0.10	0.22***	–					
OSA	0.06	0.05	0.07	−0.05	0.002	−0.11	–				
PSS	−0.31***	−0.26***	−0.35***	−0.09	−0.29***	−0.10	−0.15*	–			
PHQ-9	−0.39***	−0.31***	−0.32***	−0.14*	0.30***	−0.15*	0.02	0.60***	–		
SF-36 PCS	0.30***	0.09	0.22***	0.15*	0.01	−0.03	0.01	0.27***	−0.49***	–	
SF-36 MCS	0.34***	0.32***	0.38***	0.19***	−0.30***	0.14*	0.09	−0.64***	−0.75***	0.39***	–
Age	0.10	0.20**	0.04	−0.01	0.43***	0.10	−0.01	−0.21***	−0.29***	−0.12*	0.32***

PB, physical benefits; CB, cognitive benefits; EB, emotional benefits; SB, social benefits; TA, training age; TF, training frequency; OSA, other sports activity; PCS, physical component summary scale; MCS, mental component summary scale. *p < 0.05, **p < 0.01, ***p < 0.001.

### 3.2 Analyses

#### 3.2.1 MACS-Q factors

A confirmatory factor analysis with correlating factors was performed to validate the hypothesized four-factor model of the MACS-Q. Prior to that, assumption checks were carried out. To test the normality assumption, distribution plots were inspected, which displayed a negative skewness throughout all items. While variables with skewness < 2 and kurtosis < 2 are considered unproblematic for CFA (Bandalos and Finney, 2018), item 1 (fitness) and item 22 (satisfaction) demonstrated a kurtosis of >3, indicating potential normality issues. Therefore, Maximum Likelihood Estimation with robust error calculation was used. First, the model overall demonstrated poor fit (*χ*^2^(203) = 459.798, *p* < 0.001, *RMSEA* = 0.069, *SRMR* = 0.062, *CFI* = 0.889, *TLI* = 0.874). However, modification indices suggested corrections, which were scanned for theoretically reasonable improvements. Thus, two correlations were added between items 6 (concentration) and 8 (attention), and between items 10 (frustration) and 11 (aggression), as they conceptually overlapped within the same factor. After these modifications, the model exhibited an acceptable fit. Although the Satorra-Bentler Chi-Square was still significant (*χ*^2^(201) = 414.905; *p* < 0.001), the more recommended fit indices for larger samples improved with *RMSEA* = 0.063, *SRMR* = 0.055, *CFI* = 0.907, and *TLI* = 0.894, which suggests a reasonable fit (Bentler and Bonett, 1980; Kline, 2016). Appendix B shows the explained variances for each item by the respective factors.

#### 3.2.2 Hypotheses 1a–1d

Multiple linear regression analyses were conducted to examine the impact of training age and frequency on the perceived health benefits of MA/CS. In the first step, the control variables age and other sports activities were added. Next, either training age or training frequency was added, followed by a third step including MCS and PCS.

Partial regression plots indicated that the assumption of linearity was met for the respective outcome variable with the predictors age and training age. However, the plots showed mild floor effects with the predictors of training frequency and other sports activities, as well as strong ceiling effects with the predictors of PCS and MCS, which impeded assessing linearity for these variables. The Durbin-Watson test indicated no significant autocorrelation issues, except for the outcome variable social benefits, which showed a significant but mild deviation toward positive autocorrelation (DW = 1.74, *p* = 0.03) only in step 1. For cross-sectional data values close to 2 typically indicate negligible autocorrelation, which is why no further steps were taken to address this issue (Field, 2013). Thus, the independence of residuals could be assumed. There was no indication of multicollinearity, as all variables produced a VIF < 1.47, and tolerance statistics were > 0.68. Residuals vs. predicted plots showed a slight tendency toward linearity, which is why the assumption of homoscedasticity was not fully met. The Q-Q plots of residuals displayed a mild parabolic shape for the outcome variable, social benefits, which suggests that normality of residuals was not met for this variable. In contrast, residuals for the outcome variables, physical benefits, cognitive benefits, and emotional benefits, suggested approximate normality. As assumptions were mildly violated, bootstrapping with 1,000 replicates was performed. Overall, bootstrapped coefficients only differed minimally, suggesting that the original estimates may not have been meaningfully biased.

The results for the first two steps of the multiple linear regression are presented in Table 5. In the first step, the control variable age was significantly associated with cognitive benefits.

**Table 5:** Linear model examining predictors of perceived health benefits, with 95% bias-corrected and accelerated confidence intervals provided in parentheses.

Outcome	Step	Predictor	*b* (*95% CI*)	*BSE*	*β*	p	*aR^2^*	*ΔaR^2^*
MACS-Q PB	1	Age	0.02 (−0.001 to 0.05)	0.01	0.11	0.07	0.01	–
		Other sports activities	0.04 (−0.01 to 0.01)	0.06	0.04	0.53		
	2a	Age	−0.001 (−0.03 to 0.03)	0.01	−0.01	0.96	0.04	0.03
		Other sports activities	0.04 (−0.07 to 0.15)	0.05	0.04	0.50		
		Training age	0.05 (0.02–0.08)	0.01	0.23	0.001**		
	2b	Age	0.02 (−0.01 to 0.05)	0.01	0.11	0.08	0.01	<0.01
		Other sports activities	0.04 (−0.08 to 0.15)	0.06	0.04	0.50		
		Training frequency	0.03 (−0.01 to 0.08)	0.02	0.07	0.26		
MACS-Q CB	1	Age	0.05 (0.03–0.07)	0.01	0.22	<0.001***	0.05	–
		Other sports activities	0.09 (−0.001 to 0.003)	0.06	0.08	0.20		
	2a	Age	0.03 (−0.002 to 0.05)	0.01	0.11	0.11	0.07	0.02
		Other sports activities	0.09 (0.02–0.20)	0.06	0.08	0.18		
		Training age	0.05 (0.02–0.08)	0.02	0.21	0.003**		
	2b	Age	0.05 (0.02–0.08)	0.01	0.21	<0.001***	0.06	0.01
		Other sports activities	0.09 (−0.03 to 0.22)	0.06	0.08	0.17		
		Training frequency	0.06 (0.007–0.12)	0.03	0.13	0.04*		
MACS-Q EB	1	Age	0.01 (−0.04 to 0.06)	0.02	0.02	0.69	0.01	–
		Other sports activities	0.23 (0.02–0.43)	0.11	0.12	0.06		
	2a	Age	−0.55 (−0.11 to 0.002)	0.03	−0.14	0.05*	0.08	0.07
		Other sports activities	0.24 (0.01–0.43)	0.11	0.12	0.05*		
		Training age	0.14 (0.08–0.19)	0.03	0.32	<0.001***		
	2b	Age	0.007 (−0.04 to 0.05)	0.02	0.02	0.79	0.01	<0.01
		Other sports activities	0.24 (0.01–0.42)	0.11	0.12	0.05		
		Training frequency	0.09 (−0.01 to 0.20)	0.05	0.11	0.09		
MACS-Q SB	1	Age	0.001 (−0.02 to 0.03)	0.01	<0.01	0.98	<0.01	–
		Other sports activities	0.008 (−0.11 to 0.11)	0.06	0.01	0.90		
	2a	Age	−0.03 (−0.06 to −0.002)	0.01	−0.14	0.04*	0.05	0.05
		Other sports activities	0.01 (−0.10 to 0.13)	0.06	0.01	0.86		
		Training age	0.07 (0.03–0.10)	0.02	0.28	<0.001***		
	2b	Age	−0.001 (−0.03 to 0.02)	0.01	−0.01	0.93	<0.01	<0.01
		Other sports activities	0.01 (−0.10 to 0.13)	0.06	0.01	0.85		
		Training frequency	0.05 (−0.006 to 0.10)	0.03	0.10	0.10		

Confidence intervals and standard errors based on 1,000 bootstrap samples. Adjusted *R*^2^ is reported.

PB, physical benefits; CB, cognitive benefits; EB, emotional benefits; SB, social benefits; adjusted for age and other sports activity. *p < 0.05, **p < 0.01, ***p < 0.001.

The second step yielded significant associations of training age with all perceived health benefits. Here, training age alone was associated with physical and cognitive benefits, whereas age had an additional, albeit minor, influence on social benefits. Moreover, emotional benefits were decreasing with increasing age, while other sports activities contributed positively to the outcome. Yet, this influence of age and other sports activities disappeared with the bootstrap. For the models including training age, the explained variance ranged from 4 to 8%. When training frequency was added as a predictor in the second model, it only exhibited an influence on the perception of cognitive benefits, while age continued to show a significant influence, explaining 1% of the variance. There was no impact on the other perceived health benefits resulting from variations in training frequency.

After including training age, an additional third step revealed that the MCS was significantly associated with perceived physical benefits (*aR^2^* = 0.11, *b* = 0.04, *SE* = 0.01, *β* = 0.22, *t* = 3.21, *p* = 0.002), cognitive benefits (*aR^2^* = 0.13, *b* = 0.06, *SE* = 0.01, *β* = 0.30, *t* = 4.47, *p* < 0.001) and emotional benefits (*aR^2^* = 0.16, *b* = 0.10, *SE* = 0.02, *β* = 0.29, *t* = 4.39, *p* < 0.001). In a third step after including training frequency MCS predicted physical benefits (*aR^2^* = 0.08, *b* = 0.04, *SE* = 0.01, *β* = 0.25, *t* = 3.59, *p* < 0.001), cognitive benefits (*aR^2^* = 0.13, *b* = 0.06, *SE* = 0.01, *β* = 0.31, *t* = 4.70, *p* < 0.001) and emotional benefits (*aR^2^* = 0.12, *b* = 0.02, *SE* = 0.02, *β* = 0.33, *t* = 4.87, *p* < 0.001). In the case of cognitive benefits, the effect of training frequency was canceled out by the addition of MCS. Taken together, training age accounted for variance in all outcome variables, while frequency only showed an impact on cognitive benefits when current mental health was not taken into account.

Additionally, correlations for MACS-Q factors and training age/training frequency were calculated and are presented in Table 6. Repeating the regression analyses using the reduced 3-item physical benefits scale yielded identical patterns of effect directions and statistical significance.

**Table 6:** Spearman’s partial correlations (*ρ*) between MACS-Q factors, training age and training frequency.

Variable	Training age	Training frequency
MACS-Q PB	0.25***	0.06
MACS-Q CB	0.19**	0.14*
MACS-Q EB	0.26***	0.09
MACS-Q SB	0.29***	0.09

PB, physical benefits; CB, cognitive benefits; EB, emotional benefits; SB, social benefits; adjusted for age and other sports activity. *p < 0.05, **p < 0.01, ***p < 0.001.

#### 3.2.3 Hypotheses 2a and 2b

Partial correlations for the associations between self-rated health and perceived health benefits of MA/CS practice are presented in Table 7. Due to violations of the assumptions, Spearman’s rho was used to assess correlations. Significant positive associations were found across all benefits with MCS, with small to medium effects (*ρ* = 0.21–0.38) (Cohen, 1992). Similar effect sizes were found for the positive associations for PCS with physical, emotional, and social benefits (*ρ* = 0.15–0.31). Depression and stress were correlating negatively with physical, cognitive, and emotional benefits, displaying small to medium effect sizes (*ρ* = 0.22–0.38). Social benefits displayed a small negative relationship with depression. In summary, the correlational results indicated significant associations between self-rated physical and mental health and physical and mental health benefits.

**Table 7:** Spearman’s partial correlations (*ρ*) between perceived health benefits and health measures.

Variable	1	2	3	4	5	6	7
MACS-Q PB	–						
MACS-Q CB	0.53***	–					
MACS-Q EB	0.59***	0.63***	–				
MACS-Q SB	0.40***	0.48***	0.62***	–			
PSS	−0.29***	−0.22***	−0.34***	−0.09	–		
PHQ-9	−0.38***	−0.27***	−0.32***	−0.12*	0.59***	–	
SF-36 PCS	0.31***	0.11	0.23***	0.15*	−0.31***	−0.45***	–
SF-36 MCS	0.32***	0.27***	0.39***	0.21***	−0.61***	−0.73***	0.46***

PB, physical benefits; CB, cognitive benefits; EB, emotional benefits; SB, social benefits; adjusted for age and other sports activity. *p < 0.05, **p < 0.01, ***p < 0.001.

Given the limited internal consistency of the physical benefits subscale, these analyses were repeated using the reduced 3-item version. The pattern and significance of correlations remained unchanged.

### 3.3 Exploratory analyses

#### 3.3.1 Correlational analyses

Additional correlational analyses (Table 8), controlling for age and other sports activities, revealed a small association between training experience and perceived stress. Mental health, as measured by the PHQ-9 and MCS, showed small associations with training experience and training frequency. Correlational plots of PCS exhibited strong ceiling effects, suggesting little informative value.

**Table 8:** Spearman’s partial correlations (*ρ*) between training age, training frequency and health measures.

Variable	1	2	3	4	5	6
TA	–					
TF	0.20***	–				
SF-36 PCS	0.07	−0.02	–			
SF-36 MCS	0.19**	0.13*	0.46***	–		
PSS	−0.23***	−0.10	−0.31***	−0.61***	–	
PHQ-9	−0.21	−0.13*	−0.46***	−0.73***	0.59***	–

Adjusted for age and other sports activity; TA, training age; TF, training frequency; PCS, physical component summary scale; MCS, mental component summary scale. *p < 0.05, **p < 0.01, ***p < 0.001.

#### 3.3.2 Style-type analyses

Participants were classified as hard-style-only (*n* = 146, 54.5%), soft-style-only (*n* = 72, 26.9%), or mixed-style practitioners (*n* = 50, 18.7%). After adjusting for age, other sports activity, and self-rated physical and mental health (PCS, MCS), style type was not associated with perceived physical benefits (*aR^2^* = 0.08) and showed no clear association with cognitive benefits (*aR^2^* = 0.13). In contrast, style type was associated with emotional (*aR^2^* = 0.15) and social benefits (*aR^2^* = 0.04). Compared to hard-style practitioners, both soft-style and mixed-style practitioners reported higher emotional benefits (soft vs. hard: *b* = 2.05, *SE* = 0.77, *β* = 0.16, *t* = 2.72, *p* = 0.009; mixed vs. hard: *b* = 2.69, *SE* = 0.87, *β* = 0.18, *t* = 3.09, *p* = 0.002) and higher social benefits (soft vs. hard: *b* = 1.11, *SE* = 0.44, *β* = 0.17, *t* = 2.62, *p* = 0.009; mixed vs. hard: *b* = 1.45, *SE* = 0.49, *β* = 0.19, *t* = 2.95, *p* = 0.003). Across models, MCS was positively associated with perceived benefits (physical: *b* = 0.04, *SE* = 0.01, *β* = 0.26, *t* = 3.71, *p* < 0.001; cognitive: *b* = 0.06, SE = 0.01, *β* = 0.33, *t* = 4.87, *p* < 0.001; emotional: *b* = 0.12, SE = 0.02, *β* = 0.34, t = 5.09, *p* < 0.001; social: *b* = 0.03, *SE* = 0.01, *β* = 0.14, *t* = 1.97, *p* = 0.049). No consistent moderation effects of style type were observed. One exploratory exception was found for emotional benefits, in which the association between training frequency and emotional benefits differed between soft-style-only practitioners and hard-style practitioners (training frequency × soft-style-only: *b* = −0.24, *p* = 0.033).

#### 3.3.3 Initial training motives

Exploratory analyses examined whether initial training motives (health, fun, social contacts, self-defense, philosophy) were associated with perceived benefits and whether they moderated associations between training age and perceived outcomes. Across models adjusting for age, other sports activity, and self-rated physical and mental health (PCS, MCS), no consistent moderation effects emerged (all interaction *p*s > 0.05).

Beyond moderation, several motives showed independent associations with perceived benefits. Fun, social contact, and philosophy motives were positively associated with emotional and social benefits (*p*s < 0.05), while philosophy motivation was consistently associated with cognitive benefits (*p* < 0.001). Associations with physical benefits were smaller and less consistent.

## 4 Discussion

This study examined the perceived physical and mental health benefits of MA/CS, as well as their associations with overall wellbeing, among a broad range of MA/CS practitioners, while taking into account the influence of training age and frequency. Multiple linear regressions and partial correlation analyses were utilized to address these questions. The results suggest that training age, but not training frequency, was associated with perceived physical and mental health benefits in MA/CS practitioners. Further, the hypotheses that self-rated mental and physical health are positively associated with perceived health benefits of MA/CS can be accepted.

Research by Miyata et al. (2020) suggests that training age and training frequency of MA/CS play a role in subjective mental health outcomes (i.e., wellbeing and depression). This partially aligns with the current results, which show that training age has an impact on mental and physical health benefits. Yet, training frequency did not yield a significant impact. This may be due to the fact that the present study’s primary focus was on *perceived* health benefits, which are related to, but not identical to, self-rated health measures. Although different health questionnaires and a larger sample size were used in this study, additional correlational analyses examining the relationship between self-rated health and training age/training frequency yielded similar findings to those of Miyata et al. (2020). An additional observation is that apart from training age and training frequency, mental health (as measured by the SF-36) has a small supplementary influence on the perception of physical, emotional, and cognitive health benefits. Despite measuring a different outcome, this finding aligns with the results by Scult et al. (2017), who explored how cognitive performance relates to the emergence of depression and revealed that considering mental health at the time of assessment played a role in study results. This suggests that, similar to the present results, mental health at the time of assessment may influence the perception of health benefits. In contrast, current physical health (as quantified by the SF-36) did not influence the prediction of any outcome. This is interesting, as one would assume that worse physical health would lead to less perceived physical health benefits. There are at least three possible explanations for this. First, because strong ceiling effects occurred in the PCS, missing variability in the upper ranges might explain the failure to detect an additional influence of physical health. Second, participants may refer to longer-term outcomes when evaluating the benefits, as opposed to the shorter timeframe captured by the SF-36. Third, qualitative findings revealed that many practitioners began MA/CS for health reasons (Fuller and Lloyd, 2019). Therefore, potential health improvements may be noticed and attributed to MA/CS, even though practitioners’ physical health might be overall considered low. To minimize potential response biases based on current health, it may be valuable to further investigate how self-rated health impacts the evaluation of perceived benefits by considering this variable in future studies.

As predicted, higher self-rated physical and mental health was overall positively associated with perceived health benefits. Surprisingly, social benefits were not found to be associated with perceived stress. Since the effects were generally small, this might be the result of perceived health benefits not accurately reflecting overall wellbeing, or that the MACS-Q factor did not adequately represent social benefits. An additional explanation could be the non-normally distributed residuals for social benefits. Nonetheless, the results support the notion that health benefits are not only perceived but also reflected in participants’ health ratings.

In additional exploratory analyses, differences emerged between style types. Style was not associated with perceived physical or cognitive benefits. However, practitioners who trained in a combination of hard and soft styles reported greater emotional and social benefits than those who trained exclusively in hard styles. A similar, but weaker, pattern was observed among soft-style-only practitioners. Furthermore, exploratory analyses incorporating initial training motives as potential moderators revealed no consistent evidence that motives altered associations between training characteristics and perceived benefits, although several motives were independently associated with higher perceived emotional and social benefits. Given the cross-sectional design and exploratory nature of these analyses, the findings should be interpreted cautiously, and future research should examine style-specific and motivational mechanisms using longitudinal designs.

The factors indicate a trend for MA/CS to be perceived as beneficial, which aligns with anecdotal assertions and previous qualitative literature (Fuller and Lloyd, 2019). Interestingly, however, when compared to normative values in the healthy population, the current sample reported slightly more symptoms but did not significantly differ in perceived stress or depressive symptoms overall (Klein et al., 2016; Kocalevent et al., 2013). Additionally, Schwartz et al. (2021) found better HrQoL in Brazilian MA/CS practitioners than in the normal population using the Brazilian WHOQOL-BREF questionnaire (Fleck et al., 2000), whereas the physical and mental HrQoL as measured by the SF-36 in this study matched the German norm (Bullinger et al., 1995). Consequently, further replications with these questionnaires would be ideal to examine a possible cultural influence.

One of the strengths of this study is that it targets MA/CS-specific characteristics. This enhances the validity of the findings by ensuring contextual relevance, thereby yielding more direct practical and theoretical implications. Additionally, the robust sample size, which encompasses a diverse range of MA/CS practitioners, enhances the generalizability of the findings.

However, certain limitations have to be addressed. Some studies suggest that the relationship between health measures and training frequency is not linear, but rather decreases after reaching a certain frequency level (e.g., Jiménez-Morcillo et al., 2024; McMahon et al., 2017). This might partially explain the null findings for the influence of training frequency. Moreover, the present study did not include a distinct measure of training intensity beyond session frequency, and future research should incorporate more fine-grained indicators such as weekly training duration or perceived exertion to better differentiate between exposure and intensity effects. Similarly, it is possible that a more complex relationship exists between training experience and perceived health benefits, especially in the early stages of training, where novelty may positively affect perceived benefits (Elston, 2021). Likewise, highly experienced practitioners might attribute any perceived health benefit to their MA/CS training as a result of its deep integration into their lives. To determine if this could be verified within the current sample, the data were exploratively divided into three training age groups (see Table 1). However, the group with a training age of ≤6 months contained only a few participants (3.36%), making a meaningful comparison difficult and influencing the analyses is unlikely. Therefore, subsequent studies could further investigate this question using a larger sample size. The cross-sectional design of this study may also limit the ability to accurately depict the relationship with participants’ training age, training frequency, and perceived health benefits, as retrospective assessments do not capture real changes over time and may fail to detect a possible non-linear association. Furthermore, no assumptions about directionality of the effects can be made, and reverse causality (e.g., individuals with better health being more likely to initiate or maintain MA/CS practice) cannot be ruled out. Thus, longitudinal studies are needed to help uncover their true relationship over time and verify a causal inference.

Another constraint is that in the current sample, the MACS-Q subscale for physical benefits did not show satisfactory internal consistency, suggesting that the items may not measure a cohesive construct. Especially two items (i.e., “…my overall fitness is good”, “…I am paying attention to a healthy lifestyle”) might not be directly attributed to physical health benefits, as they explained little variance in the factor. However, excluding these items did not have a meaningful impact on the analyses. Another drawback of the MACS-Q is that it does not specify the timeframe to which it refers (e.g., “past four weeks”). Furthermore, ceiling effects in perceived health benefits throughout all domains limit the detection of actual changes. These limitations should be considered in the further development of the questionnaire by refining items and/or scoring options to ensure precision and capture nuance in the upper ranges.

Although steps were implemented to enhance robustness, the analyses may have been biased by deviations in assumptions. Additionally, selection bias may have contributed to the results, as practitioners who already held positive views on the health benefits of MA/CS might have been more likely to participate. Similarly, confirmation bias may have led practitioners to respond more favorably to questions about perceived health benefits. Future studies might address this by balancing the MACS-Q with negatively formulated items.

Finally, this study encompasses multiple commonly practiced physical activities that historically emphasize combat or self-defense, exploring the perceived health benefits on their common ground and offering a broader, more comprehensive comparison base for future research. Despite this, one might argue that the sample may still not be generalizable because not all commonly practiced MA/CS were included, and their distribution is unlikely to match the real distribution in the population. Also, the diversity of MA/CS included could itself influence the findings. For example, in a study by Görner et al. (2019), practitioners of distinct MA/CS experienced health in distinct ways. Therefore, a larger sample size would allow for direct group comparisons (e.g., “hard” vs. “soft” or “modern” vs. “traditional”) and a more nuanced picture. Considering the point in time of data collection and the limited timeframe, it was not possible to include more participants. Some club owners or associations expressed interest in the study, but cited the summer holiday season as the reason their clubs were currently closed or their members were unavailable. Therefore, future studies would ideally collect data during training seasons (i.e., fall to spring) and/or for a longer period to ensure a larger and more balanced sample. This approach would enable the inclusion of participants from smaller clubs that may be closed during the summer and also allow for a reliable identification of non-response from certain subgroups. Additionally, it is important to note that the variety of MA/CS is embedded within diverse contexts. Factors such as philosophy, teachers, training environment, and personal motivations may influence the experience of health benefits, even within a specific subcategory of MA/CS. Therefore, it would be reasonable to consider contextual factors for a more refined understanding of the influences on perceived health benefits in follow-up studies. Although the aim of this study was to explore the perceived health benefits explicitly within the group of MA/CS practitioners, comparison groups and a control group may also be of interest in further research to validate the efficacy and differentiate between sports categories and their contextual factors.

Despite these limitations, the present study makes a unique contribution to the literature by providing quantitative insights into perceived health benefits across a wide range of MA/CS, as well as the influence of training age and frequency on these perceptions. One of the main strengths of this study lies in its use of a MA/CS-specific questionnaire, which enables a direct comparison between the perceived health benefits of MA/CS and overall self-reported health. Additionally, the potential role of other sports activities was controlled to ensure the validity of the findings. The observation that training age had a small influence on perceived benefits, while the impact of training frequency was insignificant throughout most factors, suggests that the perceived health benefits of MA/CS may be similar irrespective of how long or how often the practice takes place. Given that beliefs influence our actions (Ajzen, 1991), the perceived health benefits could contribute to a commitment to MA/CS practice, even for individuals with time constraints or limited experience. Hence, emphasizing the health benefits during training when constructing MA/CS-based health interventions could be an effective way to improve mental and physical health outcomes for a broader population.

In summary, the findings demonstrate positive associations between self-rated health and perceived health benefits of MA/CS. Further, they indicate a similar perception of benefits regardless of training age or training frequency, suggesting potential health advantages for a broad range of MA/CS practitioners. However, additional research is needed to further clarify these relationships.

## Data Availability

All data used in this study are openly available on the Open Science Framework (OSF) at: https://osf.io/fc84n.
