# Supplementary material for: Perceived health benefits of martial arts and combat sports
Source: Front Psychol. 2026 Apr 20;17:1774069. doi: 10.3389/fpsyg.2026.1774069 (PMC13135931; doi:10.3389/fpsyg.2026.1774069)
Supplement: Supplementary file 1 [file Supplementary_File_1.pdf]

Appendix A: "Hard"- vs. "Soft"-Style Classifications Of Training Systems

Classification of Training Systems Into "Hard" and "Soft" Styles by Source in the Study

Category	Source in study	Training systems
"hard"-styles	Questionnaire option	Boxing; Hapkido; Karate; Kendo; Kickboxing; Krav Maga; MMA; Muay Thai; Pencak Silat; Taekwondo (TKD); Kung Fu; Wushu; Ninjutsu
	Self-added	Sami Combat Systems; Progressive Fighting System; Selbstverteidigung (Self-defense); HEMA (Historical European Martial Arts); Capoeira; Haedong Kumdo; Naginata; Wing Chun; Qwan Ki Do; Takeda Budo; Takeda Ryu; Jodo; Kobudo (Okinawan)
"soft"-styles	Questionnaire option	Aikido; Tai Chi; Judo; Brazilian Jiu-Jitsu (BJJ); Jiujitsu
	Self-added	Wrestling; Luta Livre; Qigong
Not classified	Questionnaire option	Iaido
	Self-added	Battodo

Note. Source in study indicates whether the martial art was provided as a predefined response option in the questionnaire or entered by participants in an open text field ("other training systems"). The classification into "hard" and "soft" styles follows Ciaccioni et al. (2024).
