# Supplementary material for: Perceived health benefits of martial arts and combat sports
Source: Front Psychol. 2026 Apr 20;17:1774069. doi: 10.3389/fpsyg.2026.1774069 (PMC13135931; doi:10.3389/fpsyg.2026.1774069)
Supplement: Supplementary file 2 [file Supplementary_File_2.pdf]

Appendix B: CFA Explained Variances For MACS-Q Perceived Health Benefits

R^2 *of MAQS-Q perceived health benefit items*

Item	Factor	R^2
1. fitness	Physical benefits	.06
2. immunity	Physical benefits	.38
3. injury	Physical benefits	.47
4. sleep	Physical benefits	.31
5. lifestyle	Physical benefits	.16
6. concentration	Cognitive benefits	.52
7. general cognitive function	Cognitive benefits	.59
8. attention	Cognitive benefits	.62
9. executive function	Cognitive benefits	.42
10. frustration tolerance	Emotional benefits	.35
11. aggression	Emotional benefits	.39
12. emotion regulation	Emotional benefits	.56
13. self-confidence	Emotional benefits	.25
14. self-efficacy	Emotional benefits	.56
15. anxiety	Emotional benefits	.35
16. depression	Emotional benefits	.44
17. stress	Emotional benefits	.45
18. relationships	Social benefits	.53
19. social support	Social benefits	.51
20. conflict resolution	Social benefits	.42
21. connection	Social benefits	.52
22. life satisfaction	Emotional benefits	.32
