# Supplementary material for: Perceived health benefits of martial arts and combat sports
Source: Front Psychol. 2026 Apr 20;17:1774069. doi: 10.3389/fpsyg.2026.1774069 (PMC13135931; doi:10.3389/fpsyg.2026.1774069)
Supplement: Supplementary file 3 [file Supplementary_File_3.pdf]

Online Supplement Appendix C: MACS-Q Perceived Health Benefits and Initial Motives

German Version - Perceived Health Benefits

Nun wollen wir wissen, wieviel Zusammenhang du zwischen deinem Kampfkunst- bzw. Kampfsport-Training und folgenden Aussagen siehst. Die Kampfkunst bzw. der Kampfsport trägt dazu bei, dass …

Bitte wähle, wie sehr du zustimmst. (1 = stimme überhaupt nicht zu, 2 = stimme eher nicht zu, 3 = weiß nicht, 4 = stimme eher zu, 5 = stimme sehr zu)

1. … meine allgemeine Fitness gut ist.
2. … ich selten krank bin.
3. … ich mich im Alltag kaum verletze.
4. … ich gut schlafe.
5. … ich auf einen gesunden Lebensstil achte.
6. … es für mich einfach ist, die Konzentration bei alltäglichen Dingen aufrecht zu erhalten.
7. … ich mich geistig fit fühle.
8. … ich aufmerksam bei einer Sache bleiben kann.
9. … mir das Planen und Organisieren von Projekten leichtfällt.
10. … ich durch Frustrationen im Alltag nicht so schnell aus der Fassung gerate.
11. … ich mit Wut und Aggressionen gut umgehen kann.
12. … ich unangenehme Gefühle gut meistern kann.
13. … ich mich als selbstbewusst erlebe.
14. … ich weiß, dass ich selbst schwierige Situationen im Alltag gut bewältigen kann.
15. … ich mich selten ängstlich oder nervös fühle.
16. … ich mich selten traurig oder niedergeschlagen fühle.
17. … mir Stress nicht viel ausmacht.
18. … ich gute Beziehungen zu meinen Mitmenschen pflege.
19. … ich mich durch ein soziales Netz unterstützt fühle.
20. … ich Konflikte achtsam und friedvoll bewältigen kann.
21. … ich mich verbunden mit anderen fühle.
22. … ich zufrieden mit meinem Leben bin.

German Version - Initial Motives

Wie wichtig waren für dich folgende Motive, als du mit Kampfkunst bzw. Kampfsport begonnen hast?

Bitte wähle, wie sehr du zustimmst. (1 = unwichtig, 2 = eher unwichtig, 3 = weiß nicht, 4 = eher wichtig, 5 = sehr wichtig)

1. Gesundheit
2. Spaß
3. Soziale Kontakte
4. Selbstverteidigung
5. Philosophie

English Translation - Perceived Health Benefits

We would now like to understand the extent to which you perceive a connection between your martial arts or combat sports training and the following statements. Martial arts or combat sports contribute to the fact that...
Please choose how much you agree. (1 = strongly disagree, 2 = disagree, 3 = don't know, 4 = agree, 5 = strongly agree)

1. … my overall fitness is good.
2. … I rarely get sick.
3. … I rarely injure myself in daily life.
4. … I sleep well.
5. … I pay attention to maintaining a healthy lifestyle.
6. … it is easy for me to maintain focus on everyday tasks.
7. … I feel mentally fit.
8. … I am attentive during a single task.
9. … planning and organizing projects comes easily to me.
10. … I don’t get easily upset by frustrations in everyday life.
11. … I handle anger and aggression well.
12. … I manage unpleasant emotions well.
13. … I perceive myself as self-confident.
14. … I know that I can deal with difficult situations in everyday life.
15. … I rarely feel anxious or nervous.
16. … I rarely feel sad or depressed.
17. … stress doesn’t bother me much.
18. … I maintain good relationships with others.
19. … I feel supported by a social network.
20. … I can resolve conflicts mindfully and peacefully.
21. … I feel connected to others.
22. … I am satisfied with my life.

English Translation - Initial Motives

How important were the following motives for you when you started practicing martial arts or combat sports?
Please select how much you agree. (1 = unimportant, 2 = somewhat unimportant, 3 = don't know, 4 = somewhat important, 5 = very important)

1. Health
2. Fun
3. Social contacts
4. Self-defense
5. Philosophy
